# DNA Content in Embryonic Extracellular Vesicles Is Independent of the Apoptotic Rate in Bovine Embryos Produced In Vitro

**DOI:** 10.3390/ani14071041

**Published:** 2024-03-29

**Authors:** Diego Caamaño, Joel Cabezas, Constanza Aguilera, Ioanna Martinez, Yat Sen Wong, Daniela Sanhueza Sagredo, Belén Ibañez, Sebastián Rodriguez, Fidel Ovidio Castro, Lleretny Rodriguez-Alvarez

**Affiliations:** Laboratory of Animal Biotechnology, Department of Animal Science, Faculty of Veterinary Sciences, Universidad de Concepción, Av. Vicente Mendez 595, Chillán 3780000, Chile; diecaamano@udec.cl (D.C.); joelcabezas@gmail.com (J.C.); consaguilera@udec.cl (C.A.); iomartinez@udec.cl (I.M.); ywong@udec.cl (Y.S.W.); danielasanhuezasagredo@gmail.com (D.S.S.); belenibanezjara@gmail.com (B.I.); serodriguez2017@udec.cl (S.R.); fidcastro@udec.cl (F.O.C.)

**Keywords:** EVs, DNA, bovine, embryo, apoptosis

## Abstract

**Simple Summary:**

In this study, it is described that embryos produced in vitro release nanoparticles charged with fragments of embryonic DNA. These nanoparticles, alongside their DNA content, can be recovered from the liquid medium where the embryos develop in vitro and used to assess the genetics of embryos. The analysis of gene markers is a common practice in clinics for selecting human embryos without genomic errors. In animals, it can help in selecting embryos with high genetic value. So far, this has been achieved using embryonic biopsy to collect several cells; however, this is invasive due to the manipulation of the embryo. In this sense, the secreted nanoparticles containing DNA could stand as samples for genetic analysis, without disturbing the embryo. The main concern is whether these particles are mainly released by low-quality embryos that will eventually die. This work aimed to evaluate whether the rate of cell death in the embryo affects the release of particles containing DNA. It was confirmed that viable and good-quality embryos secrete particles with DNA in the same way as low-quality embryos; therefore, they represent a suitable alternative for embryonic genetic diagnosis, avoiding the invasive nature and technical complexity of a biopsy.

**Abstract:**

Pre-implantation embryos release extracellular vesicles containing different molecules, including DNA. The presence of embryonic DNA in E-EVs released into the culture medium during in vitro embryo production could be useful for genetic diagnosis. However, the vesicles containing DNA might be derived from embryos suffering from apoptosis, i.e., embryos of bad quality. This work intended to confirm that embryos release DNA that is useful for genotyping by evaluating the effect of embryonic apoptosis on DNA content in E-EVs. Bovine embryos were produced by parthenogenesis and in vitro fertilization (IVF). On Day 5, morulae were transferred to individual cultures in an EV-depleted SOF medium. On Day 7, embryos were used to evaluate cellular apoptosis, and each culture medium was collected to evaluate E-EV concentration, characterization, and DNA quantification. While no effect of the origin of the embryo on the apoptotic rate was found, arrested morulae had a higher apoptotic rate. E-EVs containing DNA were identified in all samples, and the concentration of those vesicles was not affected by the origin or quality of the embryos. However, the concentration of DNA was higher in EVs released by the arrested parthenogenetic embryos. There was a correlation between the concentration of E-EVs, the concentration of DNA-positive E-EVs, and the concentration of DNA. There was no negative effect of apoptotic rate on DNA-positive E-EVs and DNA concentration; however, embryos of the best quality with a low apoptotic rate still released EVs containing DNA. This study confirms that the presence of DNA in E-EVs is independent of embryo quality. Therefore, E-EVs could be used in liquid biopsy for noninvasive genetic diagnosis.

## 1. Introduction

The term extracellular vesicle refers to any type of heterogenic nanoparticle population secreted or released by all cell types. These nanoparticles have a lipid bilayer surrounding molecular cargo and do not replicate because they lack a nucleus [1]. EVs are present in different body fluids, such as blood [2], urine [3], saliva [4], milk [5], amniotic fluid [6], ascitic fluid [7], endometrial fluid, [8], and bile [9]. Cells and embryos cultured in vitro also release EVs, and can thus be collected from culture media [10].

The classification criteria of EVs are described in the guidelines of the International Society of Extracellular Vesicles (ISEV). Based on these criteria, EVs are divided into two groups according to size—small vesicles (<100 nm or <200 nm diameter) and large/medium vesicles (>200 nm diameter)—to describe the isolated populations from a given fluid. However, the most common classification is based on the biogenesis of these particles, such as exosomes, which originate from multivesicular bodies (MVB), the microvesicles originating from direct plasmatic membrane evagination, and apoptotic bodies (ABs) produced by the apoptotic process [11]. Exosomes and microvesicles are released by metabolic active cells, while ABs are produced only by cells suffering from apoptosis [12]. 

EVs have been described as a mechanism of the cell to remove unnecessary or redundant proteins; however, they have an important role in cell–cell communication, mediated by the transfer of proteins, mRNAs, and microRNA to other cells [13,14]. These molecules, enclosed within a lipid membrane, are protected from degradation upon reaching close or distant cells [15]. The messages transmitted by EVs modify cellular function, participating in the control of different physiological and pathological processes, including cell proliferation, cell differentiation, cancer development, and metastasis [16]. 

EVs’ cargo differs among different populations and reflects the characteristics and function of the original cell [17]. Therefore, EVs have been proposed as disease biomarkers [18,19]. The use of released and circulating EVs for molecular diagnosis excludes the need for cellular biopsy, and is a less invasive method, often providing a more representative sample of a tissue or organ function [20]. 

Pre-implantation embryos release EVs that participate in early embryo–maternal crosstalk [10,21,22,23,24,25,26,27]. The population of embryonic EVs (E-EVs) and their concentration, size distribution, and content vary during the preimplantation period and according to embryo quality and competence [10,23,24,25,27]. E-EVs carry molecules, such as microRNAs and mRNAs, that provide information regarding the quality and signaling of the embryo and its in vitro interaction with other embryos or with endometrial/oviductal cells [24,25,28]. 

The presence of embryonic DNA in the culture media of IVP embryos is currently used in liquid biopsy for pre-implantation genetic diagnosis [29,30,31]. In a human study, the results of genetic analyses showed 30 to 90% concordance between the culture medium and embryo biopsy (reviewed by [32]). A mixture of male and female DNA in culture media from human embryos could contribute to the inconsistency of the results, probably due to the contribution of DNA from protein supplements and cumulus cells [33]. However, it seems that E-EVs also carry DNA within their molecular cargo that could be used for genetic diagnosis, increasing the specificity of the analysis by avoiding the amplification of free DNA in the culture media [34]. However, it has been difficult to explain how and why a cell releases Evs carrying genomic DNA. Thus, the E-EV population containing DNA could be derived from the apoptotic process, and the amount of DNA in the culture medium would be negatively correlated with embryonic competence [29]. 

Despite the growing interest in using the released DNA as a noninvasive method for embryo genotyping, the high rate of apoptosis that induces developmental arrest is a negative factor affecting the applicability of this method. This study aimed to evaluate the apoptotic effect of embryos on the DNA content of EVs. In conclusion, DNA fragments were found in EVs released by pre-implantation bovine embryos during in vitro culture, regardless of the embryo apoptotic rate, with this DNA identified in nanoparticle tracking analysis and the absolute DNA concentration analysis.

## 2. Materials and Methods

The procedures were carried out under the approval of the Biosafety Committee of the Faculty of Veterinary Sciences, University of Concepcion, for the execution of research project No. 1210334.

### 2.1. In Vitro Embryo Production

Bovine ovaries were collected from an abattoir and transported to the laboratory in a saline solution (0.9% *w*/*v*) at 37 °C. Cumulus–oocyte complexes (COCs) were aspirated from 3–8 mm follicles and selected based on their morphological quality. Groups of 25–30 COCs were matured in vitro (IVM) for 24 h in 4-well culture dishes (Nunc, Thermo Scientific, Waltham, MA, USA) at 38.5 °C, in an atmosphere containing 5% CO_2_ in the air. For IVM, TCM 199 Earle’s buffered medium was used, supplemented with glutamine (0.6 mM), pyruvate (0.2 mM), FSH/LH (0.2 U/mL), estradiol (1 µg/mL), gentamicin (50 µg/mL), EGF (epidermal growth factor; 10 ng/mL), and 10% (*v*/*v*) fetal bovine serum (FBS). 

### 2.2. In Vitro Fertilization Production

For the in vitro fertilization (IVF), matured COCs were incubated with thawed frozen commercial semen (Semex, Madison, WI, USA). An independent experiment assessed sperm quality using standard IVF procedures from our laboratory, confirming a favorable embryo production rate [35]. Motile sperm were selected using a Percoll gradient (45%/90%) and centrifuged at 245× *g* for 6 min. The sperm pellet was washed with 500 µL of IVF medium (TALP-IVF) and supplemented with 0.01 mg/mL heparin, 2 mM pyruvate, 50 µg/mL gentamicin, and 6 mg/mL bovine serum albumin (BSA), followed by centrifugation at 109× *g* for 3 min. IVF was performed in groups of 25–30 COCs, with a sperm concentration of 10^6^ mL. The incubation was carried out in 4-well culture dishes with 500 µL of IVF medium per well, in an atmosphere of 5% CO_2_ in air. After 18–20 h of IVF, the presumptive zygotes underwent denudation of their remaining cumulus cells through a mechanical vortex (4 min) in TCM 199-hepes with 0.3 mg/mL hyaluronidase. Zygotes were cultured for 5 d in 4-well culture dishes (25–30 zygotes per well) with 500 µL of oviductal synthetic fluid (SOF), supplemented with 0.37 mM trisodium citrate, 2.77 mM myoinositol, 10 ng/mL EGF, 2% FBS, and 3 mg/mL essentially fatty acid-free BSA at 38.5 °C, in an atmosphere of 5% CO_2_, 5% O_2_, or 90% N_2_. On Day 5, embryo development was evaluated; morulae were collected and transferred to individual culture drops of 40 µL of EV-depleted SOF medium (SOFdep) until Day 7. The culture medium was depleted of EVs by ultrafiltration of supplemented SOF using centrifugal filter devices (100 kDa, Amicon, Merck, Darmstadt, Germany) for 15 min at 1660× *g* at 4 °C. On Day 7, the embryos were classified and used for apoptosis analysis, while culture media from blastocysts and arrested morulae were collected for EV isolation and DNA analysis. 

### 2.3. Parthenogenesis Production

After oocyte maturation, the COCs were denuded with a 0.3 mg/mL hyaluronidase solution to remove the cumulus cells. Then, their activation was carried out with a first stimulus of incubation in 7% ethanol for 5 min and a second stimulus of incubation in 5 µg/mL cytochalasin B and 10 µg/mL cycloheximide for 5 h. The culture was performed as described above for IVF embryos. 

On Day 7, IVF and parthenogenetic embryos were classified as blastocysts or arrested morulae. Embryos were used immediately for apoptosis staining, and the media from blastocysts and arrested morulae were stored at 4 °C for EV isolation and DNA analysis, avoiding the thaw–freeze cycle. 

### 2.4. Terminal Deoxynucleotidyl Transferase (TdT) dUTP Nick-End Labeling (TUNEL)

For the detection of early cellular apoptosis occurring in the embryo, TUNEL analysis was performed using the In Situ Cell Death Detection Kit, Fluorescein (Roche^®^ Life Science Products; Merck, Darmstadt, Germany). First, the embryos were fixed on 4% paraformaldehyde for 1h at room temperature (RT), in an amount of 50 µL, inside a 96-well plate. Then, the embryos were washed in PBS 1×-PVP to remove the paraformaldehyde remnant. For membrane permeabilization, embryos were incubated in a solution of triton X-100 0.5% in 0.1% sodium citrate for 30 min in a wet environment. The positive and negative controls were washed, and the positive control was treated with DNAse I for 1 h at 37 °C in the dark.

All the samples, including the positive control, were stained with TUNEL dye, in amounts of 25 µL, for 1 h at 37 °C in the dark. The negative control was incubated without TUNEL dye. Finally, all the samples were incubated with Hoechst for 15 min and visualized by fluorescence microscopy. The apoptosis ratio was calculated using the number of cells positive for apoptosis and the total cell count for each embryo. 

### 2.5. Extracellular Vesicle Concentration and Characterization

Independent samples were generated for the molecular and morphological characterization of nanoparticles secreted by preimplantation bovine embryos from Days 5 to 7. For this, culture media from 100 embryos produced by IVF or parthenogenesis were collected, and one sample was pooled for each group. Nanoparticles were separated through the standard protocol described by Théry et al. [1], with modifications. To bring the pools to 15 mL, sterile PBS 1× was added. Several centrifugation steps (700× *g* for 10 min; 2000× *g* for 10 min; 10,000× *g* for 70 min) were performed to eliminate cell debris. Nanoparticles were concentrated in 20 μL using a centrifugal filter device (0.5 mL, 10 kDa, Amicon, Merck, Darmstadt, Germany) and centrifuged for 30 min at 4000× *g*. Nanoparticles were kept frozen at −20 °C until the analysis. 

For the samples used for DNA analysis, the same protocol for nanoparticle separation was used, but EVs were used immediately for the analysis.

#### 2.5.1. Nano-Tracking Analysis

A NanoSight NS300 (Malvern Instruments Ltd., Malvern, UK) was used for this analysis, equipped with a 488 nm laser and sCMOS camera, following the procedures described by Mellisho et al. [11]. An EV-depleted medium was used as a negative control. Nanoparticle characteristics, concentration, and size distribution were recorded with 20 to 100 particles per frame with a continuous flow of the samples, at room temperature (RT), using a syringe pump. 

For DNA staining of the nanoparticles, the cell membrane-permeable green nuclear dye Biotracker 488 (Merck, Darmstadt, Germany) was used with a protocol adapted from Pallinger et al. [29]. The software was configured for low particle detection and the exact dye emission. This kit has been used in other studies as a nuclear dye on cells, but is specifically for DNA and is capable of passing through the membrane. The dye was excited by a 488 nm laser and recorded using a 500 nm filter, leaving only 532 nm particle emissions for counting.

#### 2.5.2. Western Blot

Protein analysis covered membrane and cytoplasmic canonic proteins.

The samples were resuspended in a reducing buffer (0.005% bromophenol blue, 3% 2-mercaptoethanol, 9.2% SDS, 40% glycerol, and 0.5 M Tris-HCl (pH 6.8)) for 5 min at 95 °C. Proteins were separated by electrophoresis using an SDS polyacrylamide gel and transferred to a nitrocellulose membrane (Bio-Rad, Hercules, CA, USA). Thereafter, the membrane was blocked for 45 min at room temperature with 5% skim milk TBST. Next, the membranes were incubated with TSG101 rabbit (1:200 in 5% BSA + 0.5% Tween PBS E6V1X, Cell Signaling Technology, Boston, MA, USA), Alix mouse (1:1000 in 5% milk + 0.5% Tween PBS, mAb 2171, Santa Cruz, CA, USA), CD9 rabbit (1:1000 in 5% BSA + 0.5% Tween PBS, CST- D3H4P, Cell Signaling Technology, Boston, MA, USA) and APOA1 (1:1000 in 5% milk + 0.5% Tween PBS, sc-7964, Santa Cruz, CA, USA) primary antibodies at 4 °C. After overnight incubation, the membranes were incubated at room temperature for 1 h with polyclonal anti-rabbit IGG HRP conjugated (7074, Cell Signaling Technology™) or polyclonal anti-mouse IGG HRP conjugated (7076, Cell Signaling Technology™) antibodies, depending on the case. Finally, the membranes were washed thrice with TBS-T buffer (Tris–HCl, Tween 1%) and the protein signal was detected using Westar Antares ECL substrate (XLS0142, Cyanagen™, Bologna, Italy) in a GeneGnome XRQ system (Syngene™, Cambridge, UK).

#### 2.5.3. Transmission Electron Microscopy

Transmission electron microscopy (TEM) was used to identify the morphology of EVs. For the whole-mount preparations, the nanoparticles were deposited on formvar–carbon-coated copper grids and subjected to TEM analysis. The Théry et al. [1] protocol was used with slight modifications. Briefly, 10 µL of the samples was thawed and mixed with the same volume of 4% paraformaldehyde. For each group, two grids were prepared. The grids were washed and fixed with 1% glutaraldehyde and finally contrasted with uranyl oxalate solution (pH 7.0) and 4% uranyl acetate.

The grids were observed with a JEOL JEM 1200 EXII transmission electron microscope equipped with a Gatan 782 camera at the “Centro de Espectroscopía y Microscopía” (CESMI, Universidad de Concepción, Concepción, Chile). The microscope had a resolution of 5A and was operated at 80 kV. The images were obtained using the software from the equipment.

### 2.6. DNA Quantification in EVs

To determine the DNA concentration in the EVs from single embryos, a Quant-iTTM PicoGreen^®^ dsDNA Assay Kit (Invitrogen™ Waltham, MA, USA) was used. Briefly, a DNA standard curve was produced using DNA from the kit (25 ng/mL, 2.5 ng/mL, 250 pg/mL, and 0 pg/mL). Logistic regression was conducted for the identification of the sample DNA within the standard curve. Fluorescence intensity was read using the BioTek Sinergy H1 microplate reader (Agilent, Santa Clara, CA, USA) equipment. 

### 2.7. Statistical Analysis

All the statistical analyses were performed using R Studio. The effect of the technology on embryo development was evaluated using a chi-squared test. To identify the differences between the 4 groups for all the parameters analyzed, a Kruskal–Wallis test was used. For the post hoc analysis and identification of all differences between groups, a Duncan test was performed. For the correlation analysis of all the variables, a Spearman correlation test was used. Differences were considered significant at *p* < 0.05.

## 3. Results

### 3.1. Embryos with Terminal Deoxynucleotidyl Transferase (TdT) dUTP Nick-End Labeling (TUNEL) Dye and Apoptosis Determination

**Figure 1:**
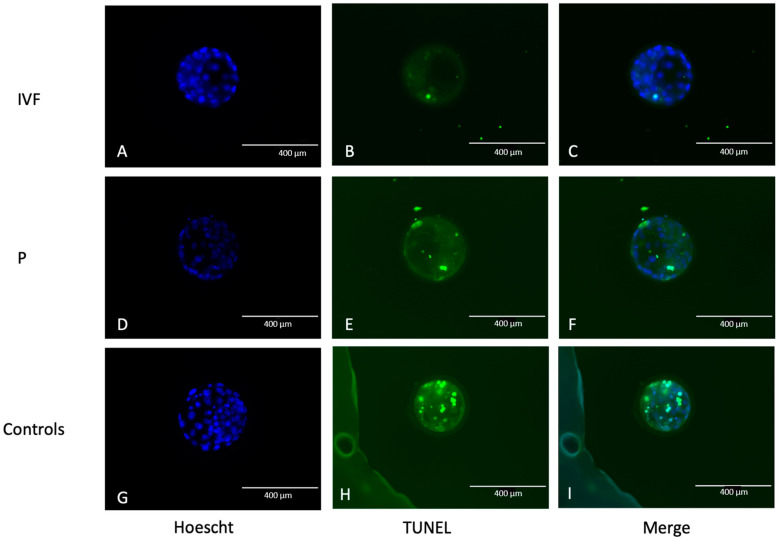
Representative image of Day 7 blastocysts with terminal deoxynucleotidyl transferase (TdT) dUTP Nick-End Labeling (TUNEL). IVF: embryos fertilized in vitro (**A**–**C**), P: parthenogenetic embryos (**D**–**F**). (**A**,**D**,**G**) Hoechst staining. (**B**,**E**,**H**) TUNEL stain. (**C**,**F**,**I**) Combination of TUNEL and Hoechst staining. Positive control with DNAse I treatment (**H**,**I**)Bars represent 400 µm.

**Figure 2:**
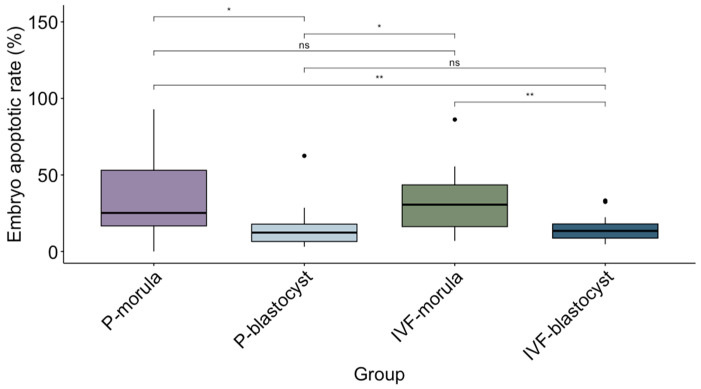
Representation of the median (line within the box) and the interquartile range of apoptotic rate in embryos from 4 different groups: P: morula and blastocyst embryos produced by parthenogenesis, and IVF: morulae and blastocysts produced by in vitro fertilization. The box represents the mean of the apoptotic rate for each group and the standard deviation (SD). Groups were compared using a non-parametric test. * represents statistical differences with *p* > 0.05. ** represents statistical differences with *p* > 0.01. ns indicates no significant differences. Black dots are outliers.

A total of 66 morulae were used to study the correlation between embryo apoptosis rate and DNA in EVs released into the culture medium. Twenty-six and forty morulae from the parthenogenetic and in vitro fertilization produced embryos, respectively. Morulae were cultured individually in EV-depleted media from Days 5 to 7 of in vitro development. Eight parthenogenetic and twenty-two IVF morulae developed to the blastocyst stage, representing blastocyst rates of 10.53% and 12.41%, respectively. The developmental capacity of parthenogenetic morulae had a tendency (*p* = 0.053) to be lower than that of IVF embryos. Though the majority of the embryos (65/66 embryos) showed apoptotic cells (Figure 1), there was no effect of the technology for embryo production on apoptotic rate. On average, the embryos had 8.01 ± 5.94 apoptotic cells and an apoptotic rate of 25.97% ± 20.55 %. Embryonic apoptosis was greater in morulae than in blastocysts, regardless of the technology for embryo production, consistent with developmental arrest (Figure 2).

### 3.2. Characterization of EVs Released by Parthenogenesis and IVF Bovine Embryos 

**Figure 3:**
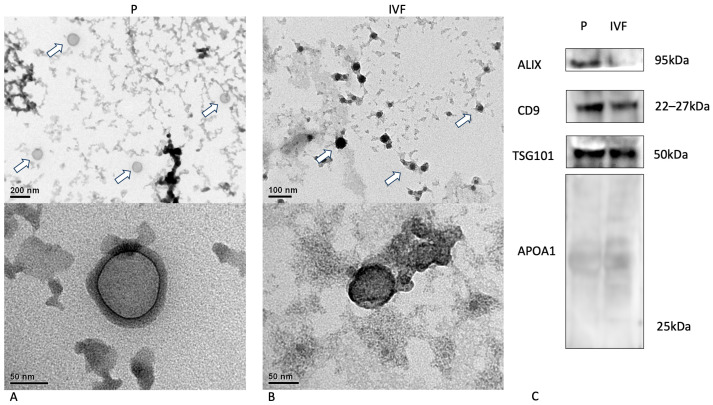
(**A**,**B**) Upper images are epresentative of EVs isolated from culture medium of embryos produced by parthenogenesis and in vitro fertilization, respectively. Arrows point to identified EVs which are enlarged in the down images. (**C**) Western Blot analysis of EV markers (ALIX, CD9, TSG101) and their purity (APOA1 absence) in both types of embryo production; P: Parthenogenesis, and IVF: embryos produced by in vitro fertilization. Western Blot (WB) results are cited in the Supplementary Materials.

Isolated embryonic EVs were characterized by their morphology, the presence of protein markers, and their size distribution and concentration. The EVs released by bovine embryos produced through parthenogenesis and IVF showed a typical morphology following transmission electron microscopy (Figure 3A,B). The classical EV surface markers Alix, CD9, and TSG101 were identified using Western blot analysis, while APOA1, a negative marker, was not detected, clearly indicating the purity of the samples (Figure 3C). 

**Figure 4:**
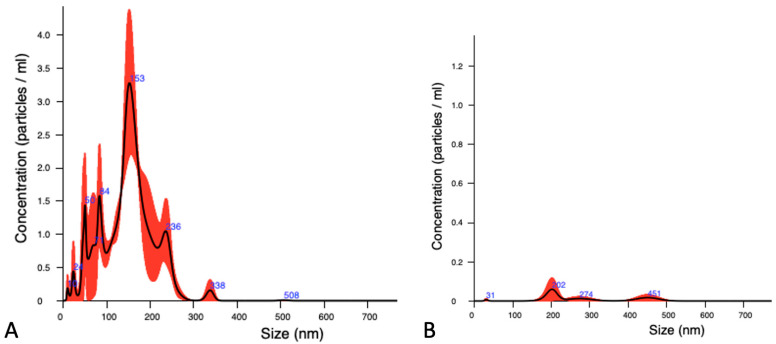
Size and distribution (*X*-axis) and concentration (*Y*-axis; (10^9^ particles × mL)) of EVs released by bovine embryos produced in vitro and parthenogenetically without DNA stain (**A**) and with DNA stain (**B**). The graphics represent the average of three technical replicates, based on the diameter and concentration distribution of nanoparticles.

EVs had a mean size of 147.62 ± 16.77 nm with an average concentration of 8.84 × 10^9^ ± 4.1 × 10^9^ particles/mL (Figure 4A). The vesicles secreted by embryos produced through IVF had the greatest size independently of the development stage. On the other hand, there was a higher concentration of particles in the samples from parthenogenetic embryos. After the staining, DNA-positive nanoparticles were detected in all samples, and a new population of big vesicles with a size over 400 nm was observed in the NTA histogram due to fluorescence (Figure 4B). A small proportion (4 ± 2.9 %) of total detected nanoparticles were positive for DNA stain. No statistical differences were observed for the concentration of DNA-positive nanoparticles among the groups (Table 1).

**Figure 5:**
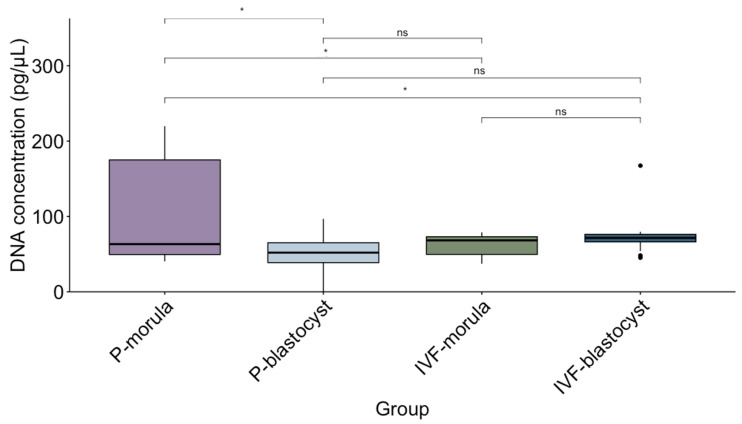
Representation of the median (line within the box) and the interquartile range of DNA concentration (pg/µL) in embryos from 4 different groups: P: morula and blastocyst embryos produced by parthenogenesis, and IVF: morulae and blastocysts produced by IVF. Groups were compared using a non-parametric test. * represents statistical differences with *p* > 0.05. ns: no significant differences.

The concentration of DNA in isolated EVs was also determined as a quantitative parameter for the analysis of its correlation with apoptotic rate in the embryos. In all samples, DNA concentration was above the baseline detected in the culture medium without embryos (13.64 ng/µL on average). The mean DNA concentrations were 62.34 ± 14.18 ng/µL and 73.50 ± 22.95 ng/µL for morulae and blastocysts produced by IVF, respectively while for parthenogenetic embryos, the values were 108.27 ± 66.82 ng/µL and 50.66 ± 28.28 ng/µL for the morulae and blastocysts, respectively. The median DNA concentration and the interquartile range for each group are presented in Figure 5. The highest DNA concentration was detected in EVs released by parthenogenetic embryos arrested at the morula stage (Figure 5).

**Figure 6:**
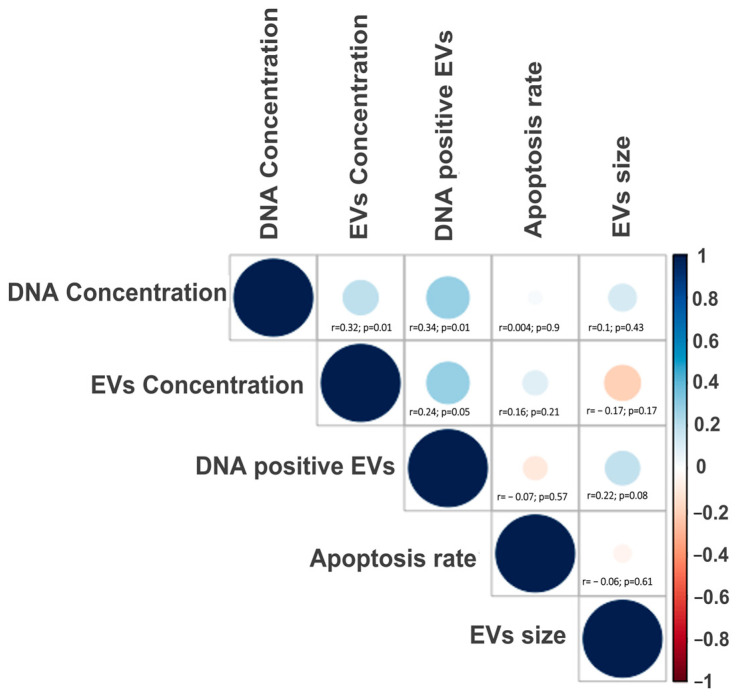
Spearman rank correlation matrix with all the variables studied from the bovine embryos experiments independent of the type of production. The graphics show a negative or positive relationship between all the variables. Blue dots represent positive correlation, while red dots indicate negative correlation. For each interception, the correlation coefficient (r) and the *p*-value are included.

A correlation analysis was performed to determine the possible effect of apoptotic rate on the embryo, the characteristics of the EV population, and the DNA content in the vesicles. For DNA content, two parameters were considered—DNA-positive EVs and DNA concentration. There was no correlation between the apoptotic rate and any of the EV parameters or DNA concentration. There was a significant and positive correlation between 1: EV concentration and DNA-positive EVs; 2: EV concentration and DNA concentration, and 3: DNA-positive EVs and DNA concentration (Figure 6). These correlations indicate that both the number of DNA-positive EVs and the amount of DNA increase with the concentration of EVs in the culture medium. In addition, when correlation analysis was performed considering the origin of the embryo and the developmental stage, there were more significant correlations for the blastocyst produced by IVF and a tendency for embryos with lower apoptotic rates to have a higher DNA-positive EV concentration (r = −0.47; *p* = 0.05). Further, the DNA concentration in EVs from IVF-derived blastocysts was independent of the apoptotic rate.

## 4. Discussion

The production of extracellular vesicles by embryos has been reported in several species, such as humans [26], pigs [28], mice [36], and bovines [10,25], with the concentration and size of the EVs varying according to embryo competence. At least in bovines, the best-quality embryos release bigger but lower concentrations of EVs during the pre-implantation period [10,23,25]. In this work, parthenogenetic and IVF embryos were compared since the first are considered to be of lower quality [37]; a similar pattern was observed for EV concentration and size. This result contributes to the cumulative data pointing to the value of EVs as markers for embryo classification.

The molecular content of embryonic EVs has been studied, with special attention to microRNAs, due to their role in the regulation of gene expression and, consequently, in cell–cell communication. The microRNA content in EVs secreted by pre-implantation embryos varies according to embryo competence, confirming that the EV cargo might reflect the characteristics of the embryo [10,25,38]. However, in addition to signaling molecules, it seems that EVs also contain DNA [39,40]. First reports showed that EVs could transport harmful DNA out of the cell. In fact, DNA is more abundant in vesicles from tumor cells compared to normal ones, and is a marker for the diagnosis of pathological conditions [41]. Thus, EVs have been related to pathological processes, also promoting tumor progression and metastasis [42,43]. 

There is much evidence indicating that EVs released by preimplantation embryos also contain DNA, which could justify the presence of DNA in the culture medium, recently used to perform PGT of IVP human embryos [29,30,31,44]. However, the published data show inconsistency in the detection and analysis of DNA in the culture medium, associated with the sensitivity of molecular techniques, the presence of DNA from other sources like cumulus cells, and the quality of the embryo [33,45]. Despite any limitations caused by the amount of DNA detected, the presence of embryonic DNA in the culture medium is very convenient for avoiding embryo biopsy for genetic analysis. The most common procedure to evaluate embryo genotyping is embryo biopsy. Cell biopsy is a complex and invasive method that involves taking a small piece of the embryo and one or more blastomeres, and it has a significant impact on the integrity of the embryo [46]. In farm animals, the use of this method is still not justified in a production program. Noninvasive methods, including liquid biopsies using embryo culture media, have been better developed for human embryos but would be useful for animal genotyping [47]. 

However, the following question arises: why would a healthy cell release DNA? Stigliani et al. [48] found a high correlation between mitochondrial DNA in culture medium and human embryo fragmentation. A report shows that the amount of DNA in the culture medium is negatively correlated with human embryo competence and clinical pregnancy [29]. A possible explanation is that DNA is free in the extracellular space or apoptotic bodies, all produced by dying cells. Apoptosis might be beneficial for the embryo during preimplantation owing to the elimination of cells with DNA damage. However, a high rate of apoptosis will eliminate a large number of cells, leading to developmental arrest [49]. Our results suggest that the apoptosis rate in embryos is higher in blocked embryos than in those that reach the blastocyst stage. These results align with other studies that evaluate apoptotic cells in bovines with competent and blocked embryos [44,50]. If the presence and quantity of DNA present in the embryo culture medium are associated with the apoptosis process, this analysis would be useful only to confirm the embryonic quality but not for genetic diagnosis, as proposed by others [51]. 

This work intended to confirm that good-quality embryos release DNA that is useful for genotyping. It is hypothesized that embryonic DNA could be released as the cargo of extracellular vesicles and that the specificity of DNA detection would increase when those vesicles are isolated from the total culture medium. DNA was detected in all EV samples. Studies on the molecular content in EVs from preimplantation embryos are limited, probably due to the sensitivity of the molecular techniques. However, to the best of our knowledge, this is the first study to detect DNA in EVs derived from bovine embryos using spectrophotometry methods or NTA analysis. The results confirm that the number of EVs containing DNA and the amount of DNA can be sufficiently detected by simple techniques, suggesting the use of EVs for genetic diagnosis in bovine embryos. 

There is a positive correlation between the number of DNA-positive vesicles and the concentration of DNA in culture media, indicating that embryonic DNA is most likely released in these vesicles. The highest DNA concentration was detected in EVs from parthenogenetic arrested morulae, which is in agreement with the finding of Stigliani et al. [48]. However, the concentration of DNA in EVs from embryos that reached the blastocyst stage was similar, independent of the embryo production technique and the number of cells suffering from apoptosis. This result confirms that competent preimplantation embryos somehow secrete DNA into the culture medium. This work did not intend to explain why a preimplantation embryo releases DNA, but is a first step to developing a noninvasive biopsy for preimplantation embryos, with a special focus on the genotyping of bovine embryos. 

## 5. Conclusions

It is concluded that EVs released during the in vitro culture of pre-implantation bovine embryos contain DNA fragments regardless of the embryonic apoptotic rate, which were identified by nanoparticle tracking analysis and absolute DNA concentration determination.

## Figures and Tables

**Table 1:** Median size and concentration of EV populations secreted by bovine embryos produced by IVF and parthenogenesis during blastulation. Total EVs and DNA-positive EVs.

Group	*n*	EVs		DNA-Positive EVs	
		Size, nm (x; IQR)	Concentration of Particles/mL (x; IQR)	Size, nm (x; IQR)	Concentration of Particles/mL (x; IQR)
Morula-P	18	143.0; 28.0 a	12.0; 4.8 × 10^9^ a	457.8; 96.2	2.8; 3.9 × 10^8^
Blasto-P	8	141.0; 12.0 a	11.1; 7.71 × 10^9^ a	513.2; 52.2	3.6; 3.4 × 10^8^
Morula-IVF	18	150.6; 21.1 b	6.0; 3.40 × 10^9^ b	478.7; 198.0	2.5; 1.7 × 10^8^
Blasto-IVF	22	154.5; 18.2 b	6.5; 3.85 × 10^9^ b	437.5; 169.9	2.3; 2.7 × 10^8^

Different letters (a and b) in the same column represent statistical differences among groups (*p* > 0.05). Comparisons were performed among groups for each parameter. X represents the median EV diameter or concentration. IQR: interquartile range.

## Data Availability

All data generated or analyzed during this study are included in this article.

## Supplementary Materials

The following supporting information can be downloaded at: https://www.mdpi.com/article/10.3390/ani14071041/s1, Western Blot (WB) results.
