# Supplementary material for: DNA Content in Embryonic Extracellular Vesicles Is Independent of the Apoptotic Rate in Bovine Embryos Produced In Vitro
Source: Animals (Basel). 2024 Mar 29;14(7):1041. doi: 10.3390/ani14071041 (PMC11011075; doi:10.3390/ani14071041)

DNA content in embryonic extracellular vesicles is independent of the apoptotic rate in bovine embryos produced in vitro

Diego Caamaño 1, Joel Cabezas 1, Constanza Aguilera 1, Ioanna Martinez 1, Yat Sen Wong 1, Daniela Sanhueza1, Belén Ibañez1, Sebastián Rodriguez1, Fidel Ovidio Castro 1 and Lleretny Rodriguez-Alvarez 1,*

	ALIX (90-100 kDa	Ponceau	ALIX/Ponceau
1	29,104,454	62,165,721	0.468175283
2	13,584,751	56,131,132	0.242018119

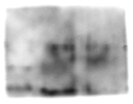

95 kDa

	CD9 (22-27 kDa)	Ponceau	CD9/Ponceau
1	38,194,295	62,165,721	0.614394788
2	32,866,546	56,131,132	0.585531501

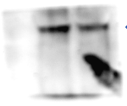

22-27 kDa

	TSG (50 kDa)	Ponceau	TSG/Ponceau
1	33,753,408	63,110,902	0.534826899
2	35,432,236	57,935,831	0.611577247

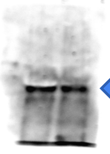

50 kDa

	APOA (28 kDa)	Ponceau	APOA/Ponceau
1	2,442,345	61,306,085	0.039838541
2	3,361,957	56,590,073	0.059408953

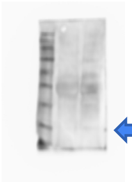

25 kDa

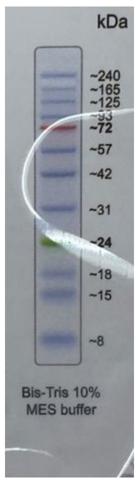

APOA - TSG/ALIX - CD9

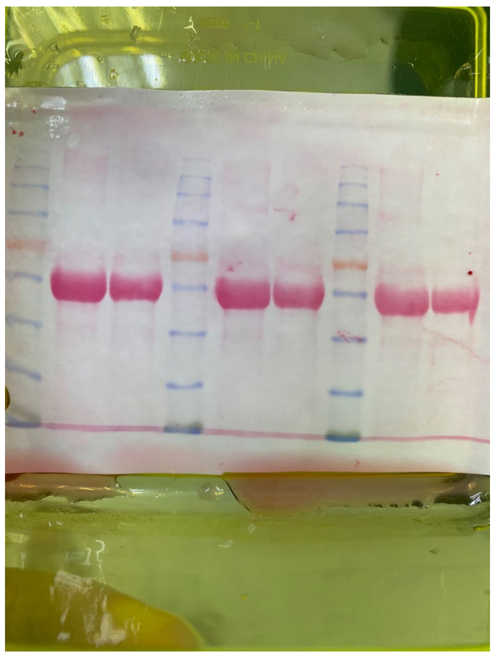

Supplement: Supplementary file 1 [file animals-14-01041-s001.zip › animals-2852061-supplementary.pdf]
